# Proteolytic Activities of Oral Bacteria on ProMMP-9 and the Effect of Synthetic Proteinase Inhibitors

**DOI:** 10.2174/1874210600802010096

**Published:** 2008-07-09

**Authors:** Guang Jie Bao, Kirsti Kari, Taina Tervahartiala, Timo Sorsa, Jukka H. Meurman

**Affiliations:** 1 Stomatology School of North-West University for Nationalities, China; 2 Institute of Dentistry, University of Helsinki, and Department of Oral and Maxillofacial Diseases, Helsinki University Central Hospital, Helsinki, Finland

**Keywords:** Oral micro-organisms, pro-matrix metalloproteinase-9, activation, proteolytic activity, synthetic proteinase inhibitors

## Abstract

Tissue reactions to bacteria lead to proinflammatory reactions involving matrix metalloproteinases (MMPs). Synthetic protease inhibitors may offer new possibilities to regulate bacterial proteases. We investigated proteolytic activities of certain periodontal bacteria, their effects on the latent proMMP-9, and the effects of synthetic MMP inhibitors and a serine protease inhibitor Pefabloc. The strains studied were *Porphyromonas gingivalis, Prevotella intermedia, Peptostreptoccus micros, Prevotella nigrescens, Fusobacterium nucleatum,* and *5 Aggregatibacter actinomycetemcomitans* serotypes. Their gelatinolytic activities and the effects of certain synthetic MMP inhibitors and Pefabloc were analyzed by zymography. Bacterial effects on proMMP-9 conversion were investigated by Western immunoblot. All investigated periodontal bacteria produced gelatinolytic cell-bound and extracellular proteinases which could fragment latent proMMP-9, suggesting co-operative processing cascades in oral tissue remodeling. A. actinomycetemcomitans produced the weakest gelatinolytic activity. Synthetic proteinase inhibitors exhibited slight but clear reductive effects on the bacterial proteolytic activities. We conclude that targeted anti-proteolytic treatment modalities against bacterial-host proteolytic cascades can be developed.

## INTRODUCTION

Tissue reaction to bacteria leads to an excessive host inflammatory response. Inflammatory cells secrete matrix-degrading proteinases, including matrix metalloproteinases (MMPs). MMPs can degrade and modify almost all matrix and basement membrane proteins in growth and in normal tissue turnover [1-2]. Elevated MMP levels, especially neutrophil gelatinase B (MMP-9) together with lesser extent neutrophil collagenase-2 (MMP-8) are considered as potential markers for tissue destruction in inflammation [1-5]. The severity and course of tissue destruction in oral infections can be monitored by assessing oral fluid MMP-8 levels [6, 7]. MMP-9 and -8 have also been shown to exert anti-inflammatory or defensive characteristics [8].

In inflammation, gelatinase B (MMP-9) is often accompanied by MMP-8 and granulocyte elastase [1-9, 10]. There is evidence that MMP-9 also increases during chronic inflammation [11]. Plasma MMP-9 seems to provide a useful marker of inflammation as it correlates with the leukocyte count and is not associated with the lipid profile [12]. Söder *et al*. [13] have shown that periodontal microorganisms can induce host response with increased release of MMP-9 and MMP-8 in the periodontal pockets. Simultaneously, higher MMP-9 concentrations were detected in blood samples of the patients indicating that this enzyme might either seep into the circulation from inflammatory cells in the periodontal pockets and/or that periodontal bacteria can trigger its up-regulation also in blood. The MMPs are synthesized as inactive proenzymes (proMMPs) and most are usually activated extracellularly to be catalytically competent. The enzyme activity is controlled by endogenous and specific tissue inhibitors (TIMPs) [14].

Periodontal bacteria, such as *Porphyromonas gingivalis, Prevotella intermedia, Peptostreptococcus micros, Prevotella nigrescens, Fusobacterium nucleatum,* and *Aggregatibacter actinomycetemcomitans*, have been identified in sub-gingival plaque as putative pathogens [15]. These and other respective species can be useful in clinical decision making especially when diagnosing and treating patients with periodontal infections [16]. Our knowledge is still sparse regarding the actual pathogenic mechanisms and interactions between bacteria and host tissue in oral infections. We set this study out to investigate the proteolytic effect of certain bacteria with special emphasis on proMMP-9 and the effects of synthetic proteinase inhibitors.

## MATERIAL AND METHODS

### Bacterial Strains, Cultivation and Sample Preparation**

Six putative periodontal pathogenic bacterial species were involved in this study. The used strains were *P. gingivalis *ATCC 33277, *P. intermedia *ATCC 25611, *P. micros *ATCC 33270, *P. nigrescens *ATCC 33563, *F. nucleatum *ATCC 25586 and 5 serotypes (a, b, c, d, e) of *A. actinomycetemcomitans *ATCC 29523 (a), ATCC 43718 (b), ATCC 33384 (c), ATCC 787 (d), ATCC 37399 (e).

The strains were grown in Brain Heart Infusion broth (BHI) (Difco Laboratories, Detroit, MI, USA) supplemented with hemin (1 mg/l) and menadione (0.5 mg/l) at 37(C to their early stationary phase, which was determined by measuring the growth curves spectrophotometrically. *A. actinomycetemcomitans *serotypes were cultivated in 5 % CO_2 _atmosphere for 8 h and the other strains anaerobically (80 % N_2_, 10 % H_2_ and 10 % CO_2_) for 48 h. Purity of each strain was monitored by Gram stain. Both cell fractions and supernatant fractions were used in this study.

The cells were harvested by centrifugation at 14,000 rpm for 20 min at 4(C, washed 3 times with neutral phosphate buffer saline (PBS) and rediluted in PBS so that the final concentrations of the cells were twice higher of the original concentration for *P. gingivalis* and 4 times higher for the other species. The cell samples were sonicated on ice until cells were disrupted. The disruption of the cells was detected by phase-contrast microscope.

After centrifugation the supernatant fractions of the growth media were collected and lyophilized to get ten-fold higher concentrations. The activity of *P. gingivalis *was high enough without concentration.

### Assay of Gelatinolytic Proteinase Activity of the Bacteria

For the measurements of gelatinolytic activity the samples of either supernatant or bacteria cells containing 5 μl of *P. gingivalis*, 8 μl of *P. intermedia*, 15 μl of *P. micros*, *P. nigrescens*, *F. nucleatum* or different serotypes of *A. actinomycetemcomitans* were incubated in dark with 5 μl of Laemmli’s sample buffer without reductant for 2 h at RT for zymography. Low range prestained SDS-PAGE standards (Bio-Rad, Hercules, CA, USA) served as molecular weight markers. Zymography with 8 % sodium dodecyl sulfate-polyacrylamide gel electrophoresis (SDS-PAGE) containing 1 mg/ml gelatin fluorescent labelled with 2-methoxy-2, 4-diphenyl-3-2H furanone (MDPF, Fluka, Buchs SG, Switzerland**)** as substrate was used. After electrophoresis, the gels were washed with Tris-HCl buffer, pH 7.5, containing 25 % Tween 80, 0.02 % NaN_3_, and then for 30 min with the same buffer supplemented with 0.5 mM CaCl_2_ and 1 μM ZnCl_2_. Finally the gels were incubated in 50 mM Tris-HCl buffer pH 7.5, containing 0.02 % NaN_3_, 0.5 mM CaCl_2_ and 1 μM ZnCl_2_ for overnight up to 7 days to detect gelatinolytic activity of proteinases. During incubation in the last buffer, different pH of 7.5, 6.5, 5.5, 4.5 and 4.0 were used to detect the optimum working condition of the bacterial gelatinolytic proteinases. The gels were monitored under UV-light within 1 to 7 days, stained with Coomassie Brilliant Blue, scanned using GS-700 Imaging Densitometer and analyzed by Quantity One –program (Bio-Rad).

### The Effects of Oral Bacteria on proMMP-9

The molecular forms of MMP-9 were detected by modified [17] Western blotting kit according to protocol recommended by the manufacturer (GE Healthcare, Amersham, UK). Aliquots of 2.5 μl (20 ng/μl) of human recombinant proMMP-9 (Invitek GmbH, Berlin, Germany) were incubated with culture media samples of *P. gingivalis*, *P. intermedia*, *P. micros, P. nigrescens, F. nucleatum,* and different serotype of *A. actinomycetemcomitans* strains at 37(C for different periods of time (2 h, 4 h, 6 h, 8 h and 24 h). The samples of cell fractions were prosessed accordingly. The same sample volumes as in zymography assay were used. Because of the strong enzyme activity of *P. gingivalis, *shorter time of incubations (0 min, 20 min, 40 min and 60 min) were also studied. For the positive control proMMP-9 was co-incubated with aminophenylmercuric acetate (APMA, Sigma, St. Louis, MO, USA) for 2h at 37^o^C. ProMMP-9 without APMA was used as negative control. The incubated samples were boiled for 5 min with the same Laemmli’s sample buffer as in zymography assay. The gels were blotted onto nitrocellulose membrane according to instructions of the manufacturer. The primary antibody used was polyclonal anti-human MMP-9 (Calbiochem, Darmstadt, Germany) and the secondary antibody anti-rabbit IgG horseradish peroxidase (1:800 dilution) (GE Healthcare). The proteins were visualized using ECL system and scanned by GS-700 Imaging Densitometer for data analysis as described above for zymograms.

### Effect of Synthetic MMP Inhibitors and Pefabloc on the Bacterial Proteinases

To determine the inhibitory effects of different synthetic MMP inhibitors on periodontal bacteria, 0.2 mM Ilomastat (ILM, Chemicon International, Inc., Temecula, CA, USA), EDTA (Merck KGaA, Darmstadt, Germany), CMT3, CMT308 (Collagenex Inc., Newtown, PA, USA), CTT1 (18)and Pefabloc (PFB, a serine protease inhibitor, Boehringer Mannheim GmbH, Mannheim, Germany) were tested in this study. The CMTs have lost their antimicrobial activity but retained their ability to inhibit mammalian MMPs [17]. Each synthetic inhibitor were dissolved in dH_2_O and diluted with TNC-buffer to final concentration of 0.2 mM. The inhibitors were incubated together with the bacterial supernatants and cell bound fractions for 2 h at 37(C in dark. The same sample volumes as in zymography assay were used.

The gelatinolytic activity was measured as previously described with zymography method using MDPF-gelatin as substrate [18, 19]. The zymograms were analyzed by Bio-Rad Model GS-700 Imaging Densitometer using the Molecular Analyst Program with correction of background values and results are expressed as arbitary units [18]. The respective samples without inhibitors were used as controls.

### Statistical Analyses

The inhibition test was repeated 3 times and the inhibition results were compared to the control data using Student’s *t* test with SPSS for Windows, version 13.0. P values less than 0.05 were considered statistically significant.

## RESULTS

### Proteolytic Activity of the Bacteria

All the putative periodontal pathogens studied showed proteolytic activity identified and measured by gelatin zymography. *P. gingivalis *yielded molecular weight bands at the area of 70-100 kDa and beyond 200 kDa. *P. intermedia, P. micros, *and *P. nigrescens *gave strong bands around 130-170 kDa. In addition, *P. intermedia* yielded a band at 60 kDa. The results were similar for both the cell supernatant or cell bound fractions. Fig. (**1**) represents proteolytic activity of cell supernatants. The cell bound fractions of *F. nucleatum* and serotypes a and d of *A. actinomycetemcomitans* did not show any gelatinolytic activity whereas serotypes b, c, and e gave the same bands as the supernatant samples (data not shown).

The pH differences in incubation buffer did not have any effect on the proteinase activities of *P. gingivalis, P. intermedia, P. micros, P. nigrescens* and *A. actinomycetemcomitans* in pH range 7.5-5.5. However, when the pH dropped to 4.5-4.0, the bands of *P. intermedia, P. micros* and *P. nigrescens* were markedly fainted (data not shown).

### The Effects of Oral Bacteria on proMMP-9

**Fig. (2):**
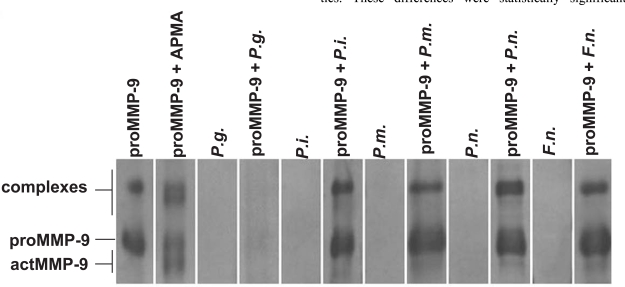
Activation of proMMP-9 by supernatants of the growth media of *P. gingivalis (P.g.), P. intermedia (P.i.), P. micros, P. nigrescens (P.n.), F. nucleatum (F.n.)* (Fig. **2**) were studied by ECL Western blot. ProMMP-9 was incubated with the supernatants at 37^o^ C for 6 h. All the strains were able to convert the 92 kDa proMMP-9 to the 60 and 77-82 kDa lower molecular size forms of MMP-9.

Western immunoblot analysis showed that supernatant of *P. gingivalis, P. intermedia, P. micros, P. nigrescens, F. nucleatum* (Fig. **2**), and *A. actinomycetemcomitans* (data not shown) growth media fragmented the 92 kDa proMMP-9 to the 60 and 77-82 kDa lower molecular species of MMP-9. The activity of *P. gingivalis *growth media was so strong that it was possible to detect even after shorter incubation times (data not shown). After 6h incubation proMMP-9 was found to be completely degraded to undetectable small peptides (Fig. **2**).

### Effects of the Synthetic MMP Inhibitors and a Synthetic Serine Proteinase Inhibitor Pefabloc on Bacterial Proteinases

The effects of *P. gingivalis, P. intermedia, P. micros, P. nigrescens* pre-incubations with ILM, EDTA, CMT3, CMT308, CTT1 and PFB on gelatin zymography are shown in Fig. (**3A** and **B**). Among all the inhibitors tested, ILM inhibited *P. micros *cell bound proteases, EDTA affected *P. gingivalis *and* P. intermedia *cell bound proteases as well as the *P. nigrescens* supernatant proteases, CMT3 inhibited *P. intermedia* cell bound proteases, CMT308 inhibited *P. gingivalis *cell bound proteases, CTT1 inhibited *P. micros* supernatant proteases and *P. intermedia *and* P. micros* cell bound proteases. Their gelatinolytic activities were reduced when compared with the respective original sample activities. These differences were statistically significant (P <0.05). Moreover, ILM affected *P. nigrescens* supernatant proteases, EDTA affected *P. nigrescens* cell bound proteases, CMT308 *P. intermedia* cell bound proteases and *P. nigrescens* supernatant proteases, CTT1 affected *P. nigrescens* supernatant proteases, and PFB affected *P. intermedia *cell bound proteases even more significantly by reducing the enzyme activities (P <0.01). In general, it seemed that all these tested synthetic inhibitors affected more effectively the bacterial cell bound proteases than the tested supernatant proteasesas shown in Fig. (**3A** and **3B**).

Because *A. actinomycetemcomitans* and *F. nucleatum* showed weak gelatinolytic activities in their supernatant samples and partly in the *A. actinomycetemcomitans* cell fractions, the effects of synthetic inhibitors were not tested for *A. actinomycetemcomitans* and *F. nucleatum.*

## DISCUSSION

The present results showed a highly complex pattern of proteinase activities of the different bacterial strains investigated which may, at least in part, indicate differences in their virulence. This was particularly clearly seen with *P. gingivalis *which is known to exert also systemic effects [26]. Our data on *A. actinomycetemcomitans* are totally new but this strain did not show strong proteolytic activity, however.

Inflammatory and immune reactions against periodontal pathogens are thought to trigger periodontal tissue destruction partly mediated by microbial proteolytic enzymes together with the host-derived MMPs [2, 20, 21]. The microbial proteinases also provide nutrients to the bacteria, in the form of small peptides and amino acids by degrading immunoglobulins, clotting factors, proteinase inhibitors and components of host connective tissue [21].

We observed that *P. gingivalis, P. intermedia, P. micros* and *P. nigrescens* not only produced proteolytic enzymes on the surface of their membranes, but also release proteinases into their surroundings. *P. gingivalis* yielded multiple molecular weight bands indicating strong proteolytic capacity in accordance with previous studies [21-22]. The major virulence factors of *P. gingivalis *include two types of trypsin-like proteinases named as ginginpains or gingivains, three types of collagenases, serine proteinase dipeptidyl peptidase and several kinds of hydrolytic proteinases [23-27]. The reported optimum pH is from 6.0-8.5, with an estimated molecular weight range from 18-300 kDa [24, 28-29]. At least three distinct collagenolytic proteinases are produced by *P. gingivalis *[30-32]. *P. gingivalis* has also shown serine dipeptidyl peptidase activity with pH optimum of 7.5-8.5. Collectively, at the periodontal lesion site, the non-restrained action of *P. gingivalis *proteinases eventually may dysregulate most mechanisms controlling inflammatory reaction of the host.

*F. nucleatum* and the five serotype strains of *A. actinomycetemcomitans* were also found to release proteolytic enzymes but with clearly less activity. Except for the sonicated cell fraction of *A. actinomycetemcomitans,* serotypes b, c, and e gave the same bands as observed in their supernatant samples. Their activities could be significantly inhibited when the pH dropped down to 4.5. However, none of the other cell preparates of *F. nucleatum* and *A. actinomycetemcomitans* showed any gelatinolytic activity. Our results about *A. actinomycetemcomitans* partly agree with and further extend those of Uitto *et al*. [33] revealing a generally low cell-associated proteinase activity, and with the results of Wang [34] by showing proteolytic activity in the supernatant samples but in different pH values and molecular weights. Furthermore, this bacterium can degrade native type I collagen and synthetic substrate for bacterial collagenases, and the activity was found both in the bacterial cellular material and in culture medium [35]. *A. actinomycetemcomitans *has shown a novel alanine- and lysine-specific peptidase activity [25-26]. It does not produce trypsin-, chymotrypsin-, elastase-, dipeptidylpeptidase- or amimopeptidase-like activities [21].

Bachrach *et al*. [36] reported that *F. nucleatum.* produced a 65 kDa proteinase which could break down extracellular matrix proteins, such as fibrinogen and fibronectin as well as type I and IV collagens. *F. nucleatum* and *P. micros* have been shown to lack trypsin-, chymotrypsin-, elastase-, dipeptidyl peptidase-, or aminopeptidase-like activity [21].

*P. intermedia* and *P.nigrescens* exhibit inhibitor-resistant dipeptidyl peptidase activity and arginine cysteine proteinase activity, which are biochemically very similar to those of *P. gingivalis *but with much weaker activities [21].

MMPs produced by inflammatory cells and tissue cells play an important role in the degradation and remodeling of extracellular matrix. In the pathogenesis of periodontal disease, tissue destruction caused by MMPs appears to be one of the pathogenic mechanisms [1-2, 37]. MMPs have been found to be processed in periodontitis-affected gingival tissues, oral fluids and even in dental plaque [5-9, 37]. Most likely MMP processing and activation *in vivo* involves co-operative action of tissue, plasma and microbial proteinases together with oxidative stress [37-38].Okamoto *et al*. [38] investigatedthe modulation potential of various bacterial proteinases againstproMMPs purified from human neutrophils (proMMP-8and -9) and from human fibrosarcoma cells (proMMP-1). Among the six differentbacterial proteinases, thermolysin family enzymes (family M4)such as *Pseudomonas aeruginosa *elastase, *Vibrio cholerae *proteinase,and thermolysin could efficiently activate all three proMMPs. Theresults indicate that certain bacterial proteinases can play an importantrole in the regulation of the tissue destruction at the site of infections [39]. Min *et al*. [40] described a novel biological activity of LPS preparations in promotion of extracellular matrix proteolysis by the degradationof proteinase inhibitor and the conversion of proMMP-9by serine proteinases trypsin and plasmin.

Seguier *et al*. [41] suggested that MMP-9 could be a marker for the clinical severity of periodontal disease. It has been suggested that proteinases expressed by the infecting periodontal pathogens might activate latent host proMMPs to initiate or accelerate degradation of the collagenous periodontal ligament [42]. Earlier studies on the activation effect of some periodontal pathogens to proMMPs mainly focused on proMMP-8 and only few investigations studied the conversion of proMMP-9 as we did in the present investigation.

Periodontal pathogens such as *P. gingivalis, A. actinomycetemcomitans* and also *Chlamydia pneumoniae* in concert with inflammatory cytokines have been reported to induce processing of the host MMPs and lead to their over-expression [43]. A proteinase from *P. gingivalis *was shown to modulate MMP-1, MMP-3 and MMP-8 and also induce secretion of collagenase fromgingival fibroblast [44]. *A. actinomycetemcomitans* has only been reported to trigger MMP-8 release and processing by human neutrophils [45], and stimulate human macrophage-like cells to secrete more MMPs* in vitro *[46].

In the present study, all the species of *P. gingivalis, P. intermedia, P. micros, P. nigrescens, F. nucleatum* and all the five serotypes of *A. actinomycetemcomitans* could process and fragment the 92 kDa recombinant proMMP-9 to its lower molecular size species, some of which corresponded to the active forms with molecular weights around 60-82 kDa [18,40]. The results on *P. gingivalis *activation of proMMP-9 are also in accordance with results of previous studies and *F. nucleatum* showed results similar to what Grenier and Grignon have reported [46]. However, the present results on *P. intermedia, P. micros, P. nigrescens *and* A. actinomycetemcomitans *strains are new and have not been reported earlier.

The possibility of synthetic proteinase inhibitors to limit or retard the bacteria-induced tissue destruction was also addressed in the present investigation. We studied the various synthetic MMPIs (ILM, EDTA, CMT3, CMT308, CTT1) and a serine proteinase inhibitor Pefabloc for their possible inhibition of proteolytic activity of the periodontal bacteria *P. gingivalis, P. intermedia, P. micros, P. nigrescens*. The results showed slight but, however, significant inhibitory effect of ILM on *P. micros* cell bound proteases, EDTA on *P. gingivalis *and *P. intermedia* cell bound proteases and on *P. nigrescens* supernatant proteases, CMT3 on *P. intermedia* cell bound proteases, CMT308 on *P. gingivalis *cell bound proteases, CTT1 on *P. micros* supernatant proteases and on *P. intermedia *and *P. micros* cell bound proteases. Furthermore, a suppressive function of their gelatinolytic activity was observed with ILM on *P. nigrescens* supernatant proteases, EDTA on *P. nigrescens* cell bound proteases, CMT308 on *P. intermedia* cell bound proteases and on *P. nigrescens* supernatant proteases, and CTT1 on *P. nigrescens* supernatant proteases. Synthetic MMP-inhibitors, either selective (CTT-1) or non-selective (ILO, EDTA, CMTs), reduce the bacterial proteinase activities in a non-specific manner and with the same efficiency [1, 19, 37]. Pefabloc showed a more pronounced effect on *P. intermedia* cell bound proteases. In general, it seemed that all these synthetic proteinase inhibitors demonstrated more efficient inhibition of cell fraction bound microbial proteinases than on proteinases released into the culture medium. Overall, our findings together with Grenier *et al*. [47] suggest that certain synthetic anti-proteolytics eventually together with some anti-microbial agents can affect the survival and proteolytic agents of periodontopathogenic bacteria.

In conclusion, the present results showed that the several periodontal bacteria investigated, i.e., *P. gingivalis, P. intermedia, P. micros, P. nigrescens, F. nucleatum* and *A. actinomycetemcomitans,* can produce cell bound and extracellular gelatinolytic proteinases which, in turn, may activate latent proMMP-9. This may suggests a co-operative cascade in the pathogenesis of oral tissue destruction. Synthetic proteinase inhibitors such as MMPIs and Pefabloc exhibit slight inhibitory effects on the proteolytic activities of these bacteria without inhibiting them completely, however. In future such synthetic inhibitors may prove to be useful tools in treatment of oral tissue destruction.

## Figures and Tables

**Fig. (1):**
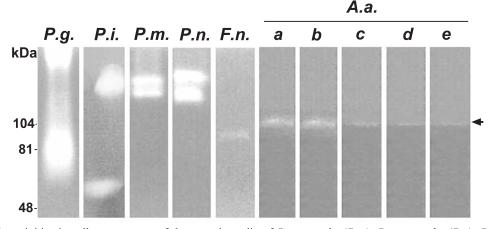
Gelatinolytic activities in cell supernatants of the growth media of *P. gingivalis (P.g.), P. intermedia (P.i.), P. micros (P.m.), P. nigrescens (P.n.), F. nucleatum (F.n.) and A. actinomycetemcomitans (A.a.)* were studied with zymographic method. The gelatinolytic activities at 103-107 kDa produced by five serotypes of A.a (a, b, c, d, e) are indicated by arrow..

**Fig. (3):**
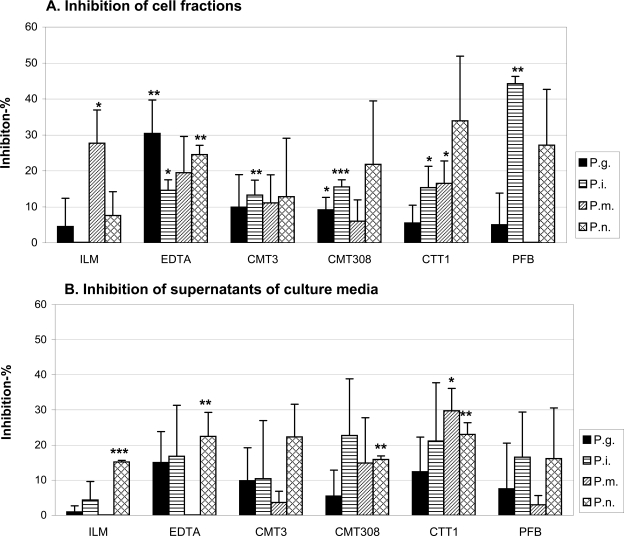
The ability of proteinase inhibitors to inhibit the bacteria-based gelatinolytic activity was studied by zymography. The test was repeated 3 times and the inhibition results were compared to the control data using Student’s t test. *  P < 0.05, * * P < 0.01,* * * P < 0.001.
